# The CUN-BAE, Deurenberg Fat Mass, and visceral adiposity index as confident anthropometric indices for early detection of metabolic syndrome components in adults

**DOI:** 10.1038/s41598-022-19343-w

**Published:** 2022-09-15

**Authors:** A. A. López-González, A. Martínez Jover, C. Silveira Martínez, P. Martínez Artal, S. Arroyo Bote, Bárbara Altisench Jané, J. I. Ramírez-Manent

**Affiliations:** 1 University School ADEMA Palma, Balearic Islands, Palma, Spain; 2 Investigation Group ADEMA-SALUD of IUNICS, Palma, Spain; 3 IDISBA, Balearic Islands Health Research Institute Foundation, Palma, Spain; 4 William Harvey Research Institute, London, UK; 5 London School of Medicine and Dentistry, Queen Mary University of London, London, UK; 6 Medical Graduate in University of Tenerife, Maxillofacial Surgery Service University Hospital of Badajoz, Badajoz, Spain; 7 Balearic Islands Health Service, Balearic Islands, Palma, Spain; 8 University Balearic Islands, Palma, Spain

**Keywords:** Cardiology, Diseases, Endocrinology, Health care, Risk factors

## Abstract

There is no definition for the metabolic syndrome; visceral obesity, elevated lipids and glucose, and hypertension coexist. The aim of the study is to determine which anthropometric indicators best determine it. Cross-sectional study in 418,343 Spanish workers. Metabolic syndrome was determined using the NCEP-ATPIII, IDF and JIS criteria. The anthropometric variables studied were: body mass index, waist circumference, waist-to-height ratio, CUNBAE, Deuremberg formula, body fat index, body surface index, normalized weight adjusted index, body roundness index, body shape index, visceral adiposity index (VAI), dysfunctional adiposity index, conicity index, metabolic score for visceral fat (METS-VF), waist triglyceride index. In men, the anthropometric indices with the largest areas under the ROC curve are VAI with ATPIII criteria and JIS. If we use the IDF criteria: waist circumference and METS-VF, with the same result. In women, the largest areas under the curve were observed with the Deuremberg formula in both ATPIII and JIS while with the IDF criteria it is METS-VF. The most useful anthropometric indices for identifying metabolic syndrome are CUN-BAE and Deuremberg, followed by the VAI. A single definition of metabolic syndrome should be agreed to determine the best anthropometric index with predictive capacity for its diagnosis.

## Introduction

Metabolic syndrome (MetS) is a complex multifactorial disorder that affects approximately 25% of people worldwide^1^ and around 30% of the adult Spanish population^2^.

The MetS encompasses a number of risk factors such as obesity, high blood pressure, dyslipidaemia, insulin resistance and endothelial dysfunction, which may lead to the development of atherosclerosis, coronary artery disease, stroke and diabetes mellitus. And is a significant risk factor for several common cancers^3^.

Identify early signs or symptoms of MetS is essential to prevent health conditions and comorbities, such as cardiovascular diseases, which remain one of the main causes of death worldwide^1^.

Among the MetS components, the blood glucose, triglycerides, and HDL-cholesterol levels require an invasive blood test. Therefore, the development and validation of a single anthropometric index for MetS with simple application and high capacity to identify early signs is of vital importance to accelerate the diagnosis and, ultimately, prevent the development of associated-diseases.

To date, the Body Mass Index (BMI) is the most widely used parameter to evaluate overweight and obesity, and is based on the relationship between weight and height. However, it does not consider lean body mass, not being effective in the evaluation of body fat or obesity^1,4^. To overcome such limitations, new anthropometric indices and lipid parameters have been developed to assess MetS in different populations.For instance, waist circumference (WC), waist-to-height ratio (WHtR), conicity index (C-Index), visceral adiposity index (VAI), body roundness index (BRI) and body shape index A (ABSI) were proposed to estimate body fat distribution^5^.

More recently, the CUN-BAE (Clinica Universitaria de Navarra Body adiposity Estimator), Deuremberg fat mass index and the, body surface index (BSI) have been suggested for the diagnosis of MetS^6^.

However, to effectively identify MetS at the earliest phase of its development or stratify people according to their real risks, it is fundamental to have a confident anthropometric index and optimal cut-off for metabolic disorders detection. In the present study, we have applied and compared 15 anthropometric indices in 418,343 workers scored for MetS diagnosis according to different International Societies.

The pathogenesis of metabolic syndrome is complex and not totally understood. Although the most accepted underlying factor is insulin resistance, there are other components such as family history, aging, hormonal disorders, environmental factors and nutrition^4^. Moreover, it is known that visceral adiposity a common feature in most of the pathogenic pathways, leading to a sustained proinflammatory and prothrombotic^7^.

## Methods

We have performed a Cross-sectional study from January 2019 to June 2020. The subjects were workers who have attended occupational medical examinations during this period. In total, 418,343 workers were included, 172,282 women and 246,061 men, from nine autonomous communities of Spain (Balearic Islands, Andalusia, Canary Islands, Valencian Community, Catalonia, Madrid, Castilla La Mancha, Castilla Leon and, Basque Country). The subjects were workers from different labour areas such as hospitality, construction, commerce, health, public administration, transport, education, industry and cleaning.

The study population was obtained from the anonymized database of workers deposited in the repository of the ADEMA-UIB university school (University of the Balearic Islands). This database comes from the occupational medical examinations carried out in the last 5 years in several occupational risk prevention services throughout Spain (RD 688/2005 of June 10 and Law 31/95 on Occupational Risk Prevention). ADEMA's anonymization system does not allow the researchers to know the identity of the workers.

### Inclusion criteria

- Age between 18 and 67 years.
- To be an active worker.
- Agree to participate in the study.

The subjects were classified and scored according to the presence of MetS using the standard definitions from:

The anthropometric measurements of height and weight as well as the, clinical and analytical data, were collected by health personnel from the occupational health units participating in the study, after standardization of the measurement techniques.

The abdominal waist circumference (WC) was measured in cm with a tape measure: *SECA* model 20, with an interval of 1–200 cm and millimetric division. For the evaluation, person was placed in a standing position, feet together and trunk erect, abdomen relaxed and upper limbs hanging down at the sides. The measuring tape was placed parallel to the floor at the level of the last floating rib^5^.

The blood pressure was determined in the supine position with a calibrated automatic sphygmomanometer OMRON M3 after 10 min of rest (cuff size adjusted to the arm circumference). Three measurements were taken at 1-min intervals and the mean of the three was calculated.

For blood analysis the subjects were asked to fast for 12 h. Blood samples were collected by peripheral venepuncture and sent to reference laboratories to be processed within 48–72 h. Glycemia, total cholesterol and triglycerides were determined by automated enzymatic methods; HDL was calculated by precipitation with dextran-sulfate Cl2Mg; LDL was estimated by the *Friedewald* formula (when triglycerides ≤ 400 mg/dL; LDL = total cholesterol − HDL − triglycerides/5). All results were expressed in mg/dL.

The following anthropometric indices and formulas were applied:

- BMI Body mass index. (BMI; in kg/m^2^), when ≥ 30 kg/m^2^ is considered obesity^5,6^.
- Waist circumference (WC)^5^. It was measured between the level of the last floating rib and the highest point of the iliac crest at the end of an exhalation.
- WtHR waist to heigh ratio^6^ = WC (cm)/height (cm).
- CUN-BAE Clinica Universitaria de Navarra Body adiposity Estimator^6^:
  $$\begin{aligned} & 44.988 + \left( {0.503 \times {\text{age}}} \right) + \left( {10.689 \times {\text{sex}}} \right) + \left( {3.172 \times {\text{BMI}}} \right) - \left( {0.026 \times {\text{BMI}}2} \right) + \left( {0.181 \times {\text{BMI}} \times {\text{sex}}} \right) \\ & \quad - \left( {0.02 \times {\text{BMI}} \times {\text{age}}} \right) - \left( {0.005 \times {\text{BMI}}2 \times {\text{sex}}} \right) + \left( {0.00021 \times {\text{BMI}}2 \times {\text{age}}} \right) \\ \end{aligned}$$
  where sex male equals 0 and female equals 1.
- Deuremberg fat mass index^6^ = 
  $$\% \;{\text{Fat}}\;{\text{mass}} = 1.2 \times \left( {{\text{BMI}}} \right) + 0.23 \times \left( {{\text{Age}}\;{\text{in}}\;{\text{years}}} \right) - 10.8 \times \left( {{\text{sex}}} \right) - 5.4$$
  where female equals 0 and male equals 1. Obesity is considered as from 25% in men and 32% in women.
- Body Fat Index.
- Body Surface Index^6^. BSA is calculated using the DuBois formula where w (weight) represents weight in kg and h (height) represents height in cm.
  $${\text{BSI}} = \frac{{{\text{WEIGHT}}}}{{\sqrt {{\text{BSA}}} }}\;{\text{and}}\;{\text{BSA}} = {\text{w}}^{0.425} *{\text{h}}^{0.725} *0.007184$$
- NWAI Normalized weight adjusted index^6^ = [(weight/10) − (10 × height) + 10], weight is expressed in kg and height in m.
- BRI Body roundness index^6^ = 364.2–365.5 × √1 − [(waist/(2π)2)/(0.5 × height)2]^5^.
- ABSI Body shape index^5^ = 
  $$ABSI \equiv \frac{WC}{{BMI^{{{\raise0.5ex\hbox{$\scriptstyle 2$} \kern-0.1em/\kern-0.15em \lower0.25ex\hbox{$\scriptstyle 3$}}}} \times height^{{{\raise0.5ex\hbox{$\scriptstyle 1$} \kern-0.1em/\kern-0.15em \lower0.25ex\hbox{$\scriptstyle 2$}}}} }}$$
- VAI Visceral adiposity index^6^ = 
  $$\begin{array}{*{20}l} \begin{aligned} {\text{Females:}}\;{\text{VAI}} & = \left( {\frac{{{\text{WC}}}}{{36.58 + (1.89 \times {\text{BMI}})}}} \right) \times \left( {\frac{{{\text{TG}}}}{0.81}} \right) \\ & \quad \times \left( {\frac{1.52}{{{\text{HDL}}}}} \right) \\ \end{aligned} \hfill & \begin{aligned} {\text{Males:}}\;{\text{VAI}} & = \left( {\frac{{{\text{WC}}}}{{39.68 + (1.88 \times {\text{BMI}})}}} \right) \\ & \quad \times \left( {\frac{{{\text{TG}}}}{1.03}} \right) \times \left( {\frac{1.31}{{{\text{HDL}}}}} \right) \\ \end{aligned} \hfill \\ \end{array}$$
- DAI Dysfunctional adiposity index^8^.
  $$\begin{aligned} & \left[ {{\text{WC}}/\left[ {22.79 + \left[ {2.68 * {\text{BMI}}} \right]} \right]} \right]*\left[ {{\text{triglycerides }}\left( {{\text{mmol}}/{\text{L}}} \right)/{1}.{37}} \right]*\left[ {{1}.{19}/{\text{high density lipoprotein}}} \right. \\ & \quad \left. { - {\text{cholesterol }}\left( {{\text{HDL}} - {\text{C}},{\text{ mmol}}/{\text{L}}} \right)} \right]{\text{ for males}}, \\ & \left[ {{\text{WC}}/\left[ {24.02 + \left[ {2.37 * {\text{BMI}}} \right]} \right]} \right]*\left[ {{\text{triglycerides}}\left( {{\text{mmol}}/{\text{L}}} \right)/{1}.{32}} \right]*\left[ {{1}.{43}/{\text{HDL}} - {\text{C}}\left( {{\text{mmol}}/{\text{L}}} \right)} \right]{\text{ for females}}. \\ \end{aligned}$$
- Conicity index^5^ = 
  $$\frac{{{\text{waist}}\;{\text{circumference}}\;({\text{in}}\;{\text{meters}})}}{0.109} \times 1/\sqrt {\frac{{{\text{weight}}\;({\text{in}}\;{\text{kilogram}})}}{{{\text{height}}\;({\text{in}}\;{\text{meters}})}}}$$
- METS-VF Metabolic score for visceral fat^9^.
  $${4}.{466} + 0.0{11}*\left( {{\text{Ln}}\left( {{\text{METS}} - {\text{IR}}} \right)} \right)^{{3}} + {3}.{239}*\left( {{\text{Ln}}\left( {{\text{WHtr}}} \right)} \right)^{{3}} + 0.{319}*\left( {{\text{Sex}}} \right) + 0.{594}*\left( {{\text{Ln}}\left( {{\text{Age}}} \right)} \right)$$
  $$METS - IR = \frac{{\ln \left[ {2*Glu\cos a\;(mg/dL) + Trigliceridos\;(mg/dL)} \right]*IMC\;(kd/m^{2} )}}{{\ln \left[ {HDL - C\;(mg/dL)} \right]}}$$
- WTG-index Waist triglyceride index^10^.
  $${\text{Waist}}\;{\text{circumference}}\;\left( {{\text{cm}}} \right) \times {\text{triglycerides}}\;({\text{mmol}}).$$

### Ethical considerations and aspects

**Figure 1:**
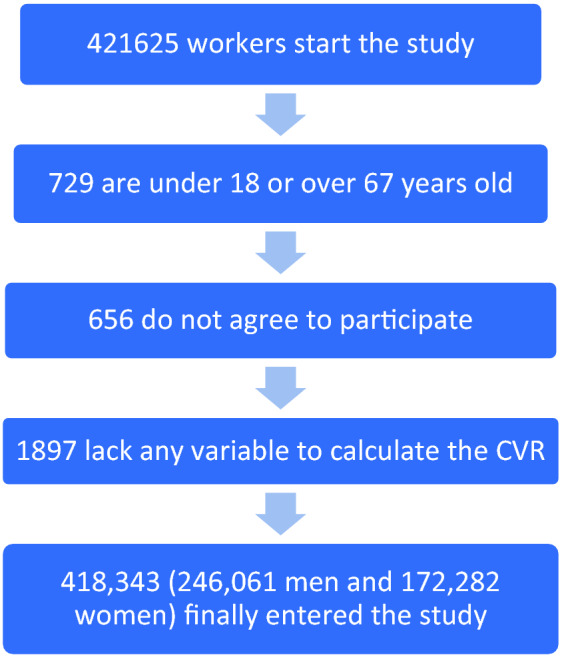
Participant flowchart.

This study was approved by the Research Ethics Committee of the Balearic Islands. All procedures were performed in accordance with the ethical standards of the institutional research committee and with the Declaration of Helsinki from 2013. All patients signed written informed consent documents before participating in the study. The participant flowchart is presented in Fig. 1.

### Statistical analysis

A descriptive analysis of the categorical variables was performed, by calculating the frequency and distribution of responses for each variable. For quantitative variables, the mean and standard deviation were calculated, whereas for qualitative variables, the test (with correction for Fisher's exact statistic when conditions required it) and Student's t test for independent samples were carried out. The Kolmogorov–Smirnov test was applied to assess the normality of the sample. To assess the usefulness of the different anthropometric indices for predicting metabolic syndrome, ROC curves are performed and the area under the curve (AUC) is determined, as well as the cut-off points with their sensitivity, specificity and Youden index. Statistical analysis was conducted with the SPSS 27.0 program, the accepted level of statistical significance being 0.05.

### Institutional review board statement

The study was carried out after the authorization of the Ethical Committee of the Balearic Islands, with the prior informed consent of the study subjects and following the norms of the Helsinki Declaration. The confidentiality of the subjects included will be guaranteed at all times in accordance with the provisions of the Organic Law 3/2018, of December 5, on the Protection of Personal Data and guarantee of digital rights and Regulation (EU) 2016/679 of the European Parliament and the Council of 27 April 2016 on Data Protection (RGPD).

### Informed consent statement

Informed consent was obtained from all subjects involved in the study.

## Results

To verify the presence of MetS among the participants of the study we have determined the predictive capacity of the different anthropometric indicators. The values of the Area Under the Curve (AUC) obtained by ROC curves were higher than 0.7 for all anthropometric indices except ABSI. For women, the ABSI index showed values lowers than 0.6 considering the three definitions of MetS (ATPIII, JIS and IDF). Similarly, the ABSI showed the lowers AUC in men classified according to all MetS definitions, (ATPIII, JIS and IDF).

The anthropometric indices with higher AUC were variable according to MetS definitions and sex. In women the most confident index was *Deuremberg* for both ATPIII and JIS MetS definitions [ATPIII = 0.907, (95% CI 0.905–0.909); JIS = 0.905, (95% CI 0.903–0.907)]. MetS classification according to the IDF the highest AUC was obtained with METSVF 0.952, (95% CI 0.951–0.953).

**Figure 2:**
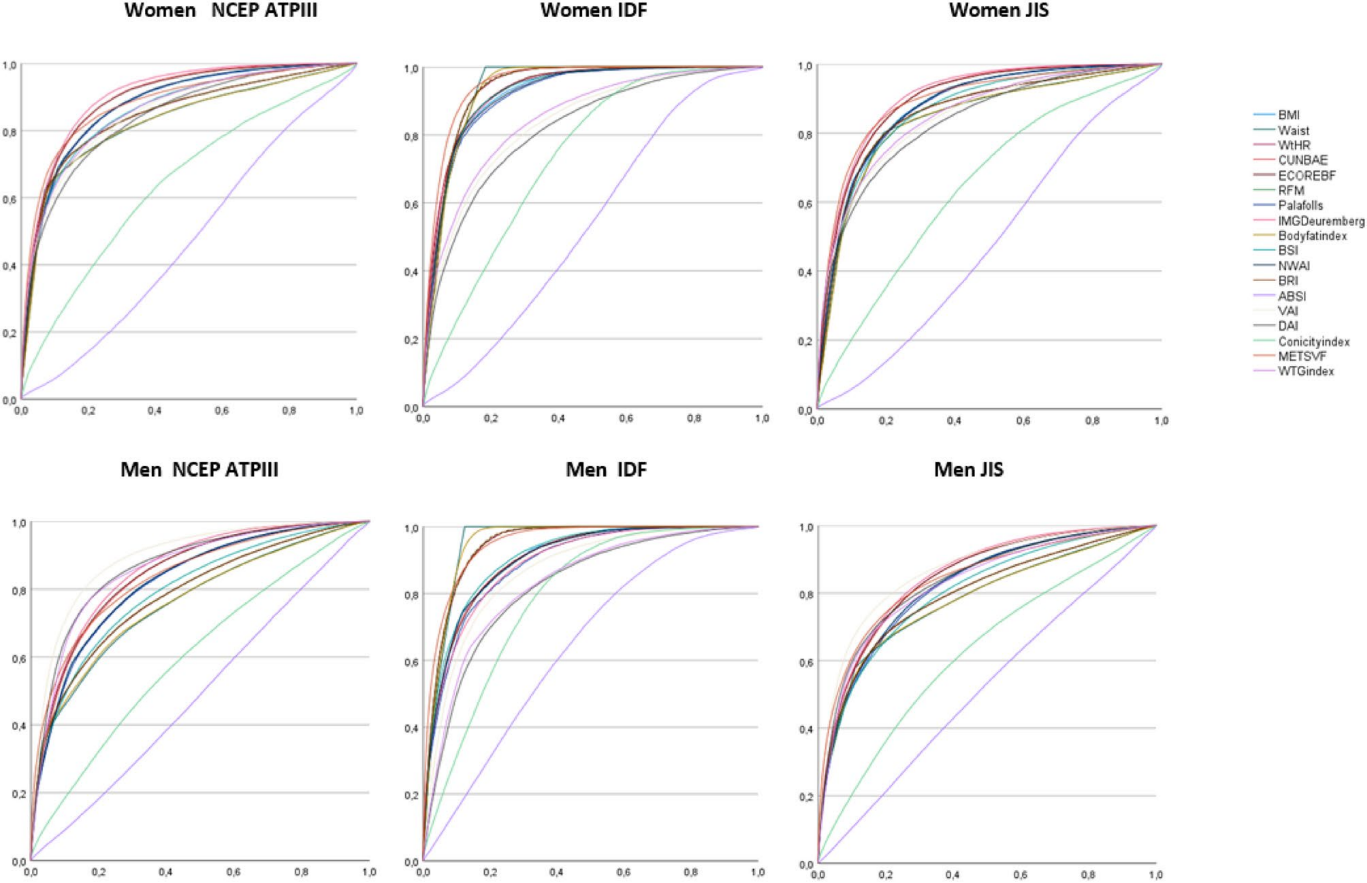
ROC curves.

For men higher AUC values were obtained with the index VAI for MetS according to ATPIII and JIS [ATPIII = 0.895, (95% CI 0.893–0.896; JIS = 0.868, (95% CI 0.866–0.869)]. While for the MetS scored according to IDF criteria the highest AUC was obtained with the WC showing a value around 1 [IDF = 0.951, (95% CI 0.951–0.952)], followed by Body fat index and METSVF with the same result [IDF = 0.950, (95% CI 0.949–0.951)]. These results can be seen in Fig. 2 (ROC curves) and Table 1 (Area under the curve (95% CI).

**Table 1:** Area under the curve (95% CI).

	Women n = 172,282			Men n = 246,061		
	NCEP-ATPIII	IDF	JIS	NCEP-ATPIII	IDF	JIS
	AUC (95% CI)	AUC (95% CI)	AUC (95% CI)	AUC (95% CI)	AUC (95% CI)	AUC (95% CI)
BMI	0.878 (0.875–0.880)	0.923 (0.921–0.924)	0.878 (0.876–0.880)	0.822 (0.819–0.824)	0.901 (0.900–0.903)	0.823 (0.821–0.825)
Waist	0.828 (0.824–0.832)	0.936 (0.934–0.937)	0.842 (0.839–0.845)	0.761 (0.759–0.764)	0.951 (0.951–0.952)	0.779 (0.777–0.781)
WtHR	0.846 (0.843–0.850)	0.936 (0.935–0.937)	0.857 (0.854–0.860)	0.783 (0.780–0.785)	0.944 (0.943–0.945)	0.795 (0.793–0.797)
CUNBAE	0.897 (0.894–0.899)	0.928 (0.927–0.930)	0.896 (0.894–0.898)	0.845 (0.843–0.847)	0.902 (0.900–0.903)	0.844 (0.842–0.845)
Deuremberg	0.907 (0.905–0.909)	0.928 (0.926–0.929)	0.905 (0.903–0.907)	0.857 (0.855–0.859)	0.890 (0.889–0.892)	0.850 (0.849–0.852)
Body fat index	0.827 (0.823–0.831)	0.933 (0.932–0.935)	0.841 (0.837–0.844)	0.763 (0.760–0.765)	0.950 (0.949–0.951)	0.779 (0.777–0.781)
BSI	0.860 (0.857–0.863)	0.922 (0.920–0.924)	0.864 (0.861–0.866)	0.794 (0.792–0.797)	0.908 (0.906–0.909)	0.803 (0.801–0.805)
NWAI	0.879 (0.876–0.882)	0.920 (0.918–0.922)	0.878 (0.875–0.880)	0.823 (0.821–0.826)	0.901 (0.899–0.902)	0.824 (0.822–0.826)
BRI	0.847 (0.843–0.850)	0.936 (0.935–0.938)	0.858 (0.855–0.861)	0.783 (0.781–0.786)	0.945 (0.944–0.946)	0.795 (0.793–0.798)
ABSI	0.475 (0.471–0.479)	0.541 (0.537–0.545)	0.481 (0.477–0.485)	0.494 (0.91–0.497)	0.643 (0.640–0.646)	0.517 (0.515–0.520)
VAI	0.856 (0.853–0.859)	0.835 (0.832–0.838)	0,848 (0.845–0.851)	0.895 (0.893–0.896)	0.866 (0.864–0.868)	0.868 (0.866–0.869)
DAI	0.842 (0.839–0.846)	0.819 (0.815–0.822)	0.834 (0.831–0.837)	0.868 (0.866–0.870)	0.821 (0.818–0.823)	0.836 (0.834–0.838)
Conicity index	0.644 (0.639–0.649)	0.738 (0.735–0.741)	0.648 (0.644–0.652)	0.604 (0.601–0.607)	0.790 (0.788–0.792)	0.625 (0.623–0.628)
METSVF	0.883 (0.880–0.886)	0.952 (0.951–0.953)	0.893 (0.890–0.895)	0.838 (0.836–0.840)	0.950 (0.949–0.951)	0.841 (0.839–0.843)
WTGindex	0.861 (0.858–0.864)	0.850 (0.847–0.853)	0.853 (0.850–0.856)	0.860 (0.858–0.862)	0.830 (0.828–0.833)	0.827 (0.825–0.829)

*NCEP-ATPIII* National Cholesterol Education Program-Adult Treatment Panel III, *IDF* International Diabetes Federation, *JIS* Join Interim Statement, *BMI* Body mass index, *WtHR* Waist to heigh ratio, *CUN BAE* Clinica Universitaria de Navarra Body adiposity Estimator, *BSI* Body Surface index, *NWAI* Normalized weight adjusted index, *BRI* Body roundness index, *ABSI* Body shape index, *VAI* Visceral adiposity index, *DAI* Dysfunctional adiposity index, *METS-VF* Metabolic score for visceral fat, *WTG-index* Waist triglyceride index, *AUC* Area under the curve.

We have applied the *Youden* index (IJ = S + E − 1) to determine the optimal cut-off point for metabolic disorders detection using the three MetS criteria.

For women scored for MetS according to the NCEP-ATPIII, the *Deuremberg* has showed a cut-off point of 38.7; with a sensitivity of 82.9% and a specificity of 82.9%, followed by CUN-BAE with a cut-off point of 40.0, sensitivity of 82.5% and specificity of 81.6%.

When using the IDF criteria to evaluate MetS, the METSVF has showed a cut-off point of 6.2 with a sensitivity of 91.0% and specificity of 87.4%, and the WC has presented a cut-off point of 81.0 with a sensitivity of 91.4% and specificity of 84.8%. Finally, classifying MetS according to the JIS criteria, again the *Deuremberg* and METSVF showed best confidence. *Deuremberg* presented a cut-off point of 38.3, sensitivity of 82.8% and specificity of 82.7%, and showed a cut-off point of 6.0, sensitivity of 83.7% and specificity of 81.6% as can be seen in Table 2. When analysing the anthropometric indices applied to men the cut-off points were also variable according to MetS definitions. For MetS classification according to NCEP-ATPIII, the VAI has showed a cut-off point of 8.1, sensitivity of 82.3% and specificity of 82.2%, and the WTG index showed a cut-off point of 132.9 with a sensitivity of 79.7% and specificity of 79.5%. While for MetS classification according to the IDF, the WC has presented the most confident cut-off point of 94 with a sensitivity of 95.6% and specificity of 88.4%, followed by the Body fat index cut-off of 28.3 with a sensitivity and specificity of 89.3%. Lastly, according to the JIS criteria, the BRI has showed a cut-off point of 3.5 with a sensitivity of 73.2% and specificity of 72.9%, followed again by the VAI index with a cut-off point of 6.9 and sensitivity and specificity similar to those found previously with the NCEP-ATPIII criteria for MetS classification (82.3% and 82.2%, respectively) as shown in Table 3.

**Table 2:** Cut-off points, sensitivity, specificity and Youden index in women.

	Women n = 172.282		
	NCEP-ATPIII	IDF	JIS
	Cutoff–Sens–Specif–Youden	Cutoff–Sens–Specif–Youden	Cutoff–Sens–Specif–Youden
BMI	27.5–81.6–78.6–0.6	28.6–85.1–84.8–0.6	27.5–80.8–79.6–0.6
Waist	78.0–77.4–74.3–0.5	81.0–91.4–84.8–0.7	79.0–80.9–79.2–0.6
WtHR	0.4–78.9–77.1–0.5	0.50–91.1–85.0–0.7	0.5–80.3–80.3–0.6
CUNBAE	40.0–82.5–81.6–0.6	41.0–85.6–85.6–0.7	39.7–82.4–82.4–0.6
Deuremberg	38.7–82.9–82.9–0.6	39.2–85.2–85.2–0.7	38.3–82.8–82.7–0.6
Body fat index	29.5–76.2–76.2–0.5	32.1–86.5–86.4–0.7	29.9–71.2–71.2–0.4
BSI	54.0–78.7–78.1–0.5	56.0–85.6–85.4–0.7	54.0–79.1–78.9–0.5
NWAI	1.1–81.4–78.9–0.6	1.2–86.0–83.5–0.6	1.1–80.3–79.8–0.6
BRI	3.1–78.8–77.4–0.5	3.5–87.4–87.3–0.7	3.1–80.1–80.1–0.6
ABSI	0.1–54.4–49.4–0.1	0.1–55.3–49.4–0.1	0.1–53.2–49.2–0.0
VAI	3.1–77.6–77.4–0.5	3.0–75.7–75.6–0.5	3.0–76.7–76.6–0.5
DAI	0.7–76.2–76.2–0.5	0.7–74.5–74.0–0.4	0.7–76.5–74.1–0.5
Conicity index	1.1–61.3–61.3–0.2	1.1–66.7–66.5–0.3	1.1–61.1–61.1–0.2
METSVF	6.1–81.7–80.9–0.6	6.2–91.0–87.4–0.7	6.0–83.7–81.6–0.6
WTGindex	86.1–78.9–77.4–0.5	86.0–77.0–76.9–0.5	85.0–77.4–77.2–0.5

*NCEP-ATPIII* National Cholesterol Education Program-Adult Treatment Panel III, *IDF* International Diabetes Federation, *JIS* Join Interim Statement, *BMI* Body mass index, *WtHR* Waist to heigh ratio, *CUN BAE* Clinica Universitaria de Navarra Body adiposity Estimator, *BSI* Body Surface index, *NWAI* Normalized weight adjusted index, *BRI* Body roundness index, *ABSI* Body shape index, *VAI* Visceral adiposity index, *DAI* Dysfunctional adiposity index, *METS-VF* Metabolic score for visceral fat, *WTG-index* Waist triglyceride index.

**Table 3:** Cut-off points, sensitivity, specificity and Youden index in men.

	Men n = 246.061		
	NCEP-ATPIII	IDF	JIS
	Cutoff–Sens–Specif–Youden	Cutoff–Sens–Specif–Youden	Cutoff–Sens–Specif–Youden
BMI	27.8–74.8–74.5–0.4	28.7–82.0–81.3–0.6	27.2–74.8–74.7–0.4
Waist	88.0–71.8–66.0–0.3	94.0 95.6–88.4–0.8	87–73.6–67.6–0.4
WtHR	0.5–74.2–67.2–0.4	0.5–92.4–84-0.7	0.5–75.0–70.0–0.4
CUNBAE	28.2–76.6–76.5–0.5	29.4–82.0–82.0–0.6	27.2–76.3–76.3–0.5
Deuremberg	27.8–77.7–77.6–0.5	28.6–80.6–80.6–0.6	26.7–77.0–77.0–0.5
Body fat index	24.0–69.8–69.4–0.3	28.3–89.3–89.3–0.7	23.3–71.6–71.6–0.4
BSI	59.8–72.2–72.2–0.4	61.9–82.7–82.7–0.6	58.9–73.0–72.9–0.4
NWAI	1.0–75.9–73.4–0.4	1.3–83.4–80.0–0.6	0.8–75.1–74.5–0.4
BRI	3.6–71.3–71.1–0.4	4.1–87.7–87.6–0.7	3.5–73.2–72.9–0.6
ABSI	0.1–50.6–48.5–(– 0.1)	0.1–60.0–60.0–0.2	0.1–53.9–49.5–0.1
VAI	8.1–82.3–82.2–0.6	8.1–79.3–79.0–0.5	6.9–79.2–79.0–0.5
DAI	0.9–79.8–79.2–0.5	0.9–75.2–75.1–0.5	0.8–76.1–76.0–0.5
Conicity index	1.1–62.6–62.5–0.2	1.2–71.6–71.5–0.4	1.1–64.7–64.7–0.2
METSVF	6.6–76.1–75.4–0.5	6.8–88.2–88.2–0.7	6.5–76.9–76.9–0.538
WTGindex	132.9–79.7–79.5–0.5	131.2–75.6–75.6–0.5	116.2–75.5–75.4–0.5

*NCEP-ATPIII* National Cholesterol Education Program-Adult Treatment Panel III, *IDF* International Diabetes Federation, *JIS* Join Interim Statement, *BMI* Body mass index, *WtHR* Waist to heigh ratio, *CUN BAE* Clinica Universitaria de Navarra Body adiposity Estimator, *BSI* Body Surface index, *NWAI* Normalized weight adjusted index, *BRI* Body roundness index, *ABSI* Body shape index, *VAI* Visceral adiposity index, *DAI* Dysfunctional adiposity index, *METS-VF* Metabolic score for visceral fat, *WTG-index* Waist triglyceride index.

## Discussion

We have evaluated the predictive capacity of anthropometric indicators for the diagnosis of MetS through the use of a large database from the ADEMA-UIB university school. This has allowed us to analyze a sample of 418,343 workers, 172,282 women and 246,061 men. The predictive performance of the indices for identifying MetS was compared using receiver operating characteristic (ROC) curves and areas under the curves (AUC).

As there is still no consensus on a MetS definition, we used the three main definitions: NCEP/ATP-III, JIS and IDF^8^. The latest guidelines recognize metabolic syndrome as a set of laboratory and anthropometric abnormalities, in which the patient presents at least three altered parameters to be diagnosed as metabolic syndrome. These parameters are: increased waist circumference, high triglyceride values or being on drug treatment, low HDL levels, arterial hypertension and fasting blood glucose ≥ 100 mg/dL or being treated for diabetes mellitus^11^. In our study we assessed as MetS the presence of 3 or more of the above parameters altered for any of the three definitions of MetS.

AUC were developed using binomial logistic regression analysis, with MetS (Yes = 1, No = 0) as the response variable and anthropometric parameters as predictors. In most of the results obtained, the AUC found a value greater than 0.75 for both men and women, except for the Conicity and ABSI index, which reflects a good test value.

Among the variables according to sex, *Deuremberg* obtained the highest AUC according to both ATPIII (0.907) and JIS (0.905), and METSVF (0.952) for IDF in the case of women. While in men, VAI had the highest AUC according to ATPIII (0.895) and JIS (0.868), and WC (0.951) in the case of IDF.

In a study in Taiwan^12^ conducted on a sample of 5000 individuals from the Taiwan Biobank database. They used the National Cholesterol Education Program Adult Treatment Panel III (NCEP/ATP-III) definition of MetS, which is used by the Taiwan Health Promotion Administration. This study evaluated the predictive capacity and the cut-off value of 11 parameters related to obesity. Finding that TyG index (triglyceride glucose index) and VAI may be the relevant indexes to evaluate MetS in clinical practice. ROC curve analysis showed that VAI had the highest AUC in men (AUC = 0.867) and women (AUC = 0.925) aged 30–50 years, whereas TyG index had the highest AUC in men (AUC = 0.849) and women (AUC = 0.854) aged 51–70 years^12^.

In our study we have not evaluated the TyG, so we cannot make a comparison with it; in addition, we have not stratified by age, which may produce differences with the results of the aforementioned study. In our work, the VAI also obtained good results in the analysis of the ROC curves with an AUC in men (NCEP-ATPIII = 0.895, IDF = 0.866, JIS = 0.868) and in women the AUC was (NCEP-ATPIII = 0.856, IDF = 0.835, JIS = 0.848). The sensitivity and specificity analysis, the three formulas present a *Youden* index greater than 0.5 in women with a sensitivity and specificity greater than 75%.

For men, the *Youden* index is also greater than 0.5 with all three formulas approaching 0.6 with a sensitivity and specificity of more than 79%. Therefore, our results would be in agreement with those of Chiu et al., in that the VAI may be a good useful index to identify MetS in adults.

In the meta-analysis by Bijari et al.^13^, of bivariate diagnostic test accuracy analysis, the AUC of the ROC curves was 0.847, indicating a moderate to high detection accuracy of VAI for MetS, with a combined sensitivity of 78% and specificity of 79%, which agrees with the previous study and our results.

A study conducted in Korea^14^ on a sample of 134,195 people from the Health Examinees Study (HEXA) which is part of the Korea Genome Epidemiology Study (KoGES)^15^. In this case, the VAI is not found among the anthropometric indices evaluated. Of the indices evaluated in this study, both in men and women, the greather AUCs for discriminating metabolic abnormalities were obtained for the waist-to-height ratio (AUC [95% confidence intervals], 0.677 [0.672–0.683]) in men and (0.691 [0.687–0.694]) in women. In our study, the waist-to-height ratio AUC greater than 0.7 in men for the three formulas studied and AUC greater than 0.8 in women also obtained good results.

A cross-sectional study by Wu et al.^5^, has comprehensively compared the ability of 10 anthropometric indices to identify MetS. In this study MetS was defined according to the International Diabetes Federation (IDF) criteria definition. Among the ten indices analyzed, AVI and WHT.5R show superior ability to identify MetS. WHT.5R has not been evaluated in our study so we cannot establish comparisons. In the case of AVI, for men it tended to be a more useful predictor for MetS (AUC: 0.767, sensitivity 79.6%, specificity 69.7%, and for women, AVI had the greather predictive value for MetS (AUC: 0.749, sensitivity 85.4%, specificity 65.2%.

In the second stage of the Bushehr Elderly Health (BEH) program in Iran^16^, a cross-sectional study was conducted based on data from 2426 adults aged ≥ 60 years who participated in the aforementioned study. MetS was defined based on the revised National Cholesterol Education Program Adult Treatment Panel III (NCEP-ATP III) criteria. Seven antrometabolic indices were studied to assess the predictive performance for identifying MetS, using receiver operating characteristic (ROC) curve analysis. Logistic regression analysis was applied to determine associations between MetS and indices. In the general population, VAI and LAP had the highest predictive power for MetS with AUC 0.87 (0.86–0.89) and 0.87 (0.85–0.88), respectively.

Another study conducted in Iran by Baveicy et al. ^17^ on a sample of 10,000 people aged 35–65 years and the IDF definition of MetS was used. The results of this study conclude that of the 3 anthropometric indices studied VAI is the best predictor of MetS with an AUC 0.86 in women (95% CI 0.85–0.87) and of 0.82 in men (95% CI 0.81–0.83).

Multiple studies have sought to determine the best anthropometric index to predict MetS conclude that the best index is VAI^18–20^. Although there are some publications with different results while new anthropometric indices appear as predictors of MetS.

The lack of consensus on the MetS diagnostic criteria is a very important issue to determine the best indicator. Since, due to the differences in the standard criteria (ATP III, IDF, JIS) used for the diagnosis of MetS and discrepancies in cut-off values, it is often difficult to compare the results obtained^8^.

In our study we wanted to analyze each of the anthropometric indices analyzed with the three definitions of MetS (ATP III, IDF, JIS). Our results agree with previous studies in that VAI is a good predictor for MetS, both in men and women. The results obtained in our study show us an area under the curve greater than 0.8 for both sexes with the three definitions used (Table 1).

The optimal cut-off points presented a Youden index greater than 0.5 in the three formulas used in both sexes. These results show that based on AUC, sensitivity and specificity, VAI is a good predictor for MetS (Tables 2, 3), this is in part due to the high degree of correlation of the measurement with TG and HDL-C.

However, in our study we have evaluated other anthropometric indices for the diagnosis of MetS for which we have not found literature to make comparisons with. We have evaluated the CUMBAE and the Deuremberg, whose formulas take into account the sex and age variables for the calculation of obesity.

In both cases and with the three MetS definitions used, the area under the curve was higher than 0.8 in both men and women, which shows a good result.

In women, the results obtained according to the Deuremberg formula show an AUC higher than 0.9, which is higher than those obtained with the VAI. This indicates that the test offers good diagnostic performance with a very narrow confidence interval. The Youden index is also high in all cases, exceeding 0.65 with high sensitivity and specificity. This strengthens the usefulness of the test.

The results obtained with the CUN-BAE are similar, with an AUC higher than 0.89 in the three formulas used and a very narrow confidence interval. The Youden index presents a result which is similar to the one obtained with the CUN-BAE with a value of 0.64 or higher, which summarizes a good performance of the diagnostic test.

This reflects better results than those obtained with the VAI. By obtaining AUC closer to the unit and a Youden index greater than 0.64 in both cases and for the three formulas, indicating a higher specificity and sensitivity.

In the case of men, when using the Deuremberg and CUN-BAE formulas, the results are also positive, with an AUC greater than 0.84 in the three models for determining MetS and a Youden index greater than 0.5.

These results, despite being favorable, are lower than those obtained in women for the three MetS models and the two formulas (CUN-BAE and Deuremberg). This is different from the VAI formula in which the AUC for the three MetS models is somewhat higher in men.

When we compare the results obtained between VAI, CUN-BAE and Deuremberg, it can be noted that the results of the last two formulas exceed those obtained in the VAI formula in women and hardly present differences when comparing with men. Although the CUN-BAE and Deuremberg formula are based on BMI, they have the advantage that they also take into account the age and sex of the subjects. In the study by Vinknes et al. they also found that CUN-BAE is better than BMI for the prediction of MetS^21^, as also seen in other works by different authors^22–24^. Other studies conducted in patients with intermediate cardiovascular risk found no differences between CUN-BAE and the other formulas analyzed^25^.

When we assess the AUC for the different formulas used, we find that in most of them it is greater than 0.75, both in men and women, with the exception of the ABSI and the Conicity index. This indicates that the test is good in the rest of the formulas used.

CUN-BAE and Deuremberg are formulas that take into account differences in adiposity according to age and sex. It is known that women and older people have a higher percentage of body fat with the same BMI. Aging causes many changes in body composition, increasing body fat, decreasing water content, often without producing changes in BMI; in fact, as the individual ages, the amount of fat increases and the lean tissue or muscle mass decreases, at the same time the lipids are introduced into other tissues such as the liver. This can affect on the methods to assess body composition^26^.

The VAI includes three parameters (TG, HDL-C and BMI) that are part of the three MetS definitions used in our study, these factors influence the predictive accuracy in different MetS criteria. Therefore, it is to be expected that it is a good index for the detection of MetS, as has been shown in other studies ^12,13,16,17^.

In the present study, VAI, CUN-BAE, and Deuremberg exhibited higher AUC in MetS identification than other traditional obesity-related formulas.

Although the AUC of most of these formulas are higher than 0.75 (good test), in several of the results obtained with the Deuremberg and CUN-BAE formulas the result is higher than 0.9 (very good). The Youden index, summarizes the performance of a diagnostic test, to the AUC result. In the study of the Deuremberg and CUN-BAE formulas, we found an index of 0.64 or higher in the three ways of evaluating MetS in women, which does not happen with the other formulas. In men, the Youden index is 0.52 or higher in the three methods of evaluating MetS, and it is below 0.4 in many of the results of other formulas.

This study indicated that both CUN-BAE and Deuremberg formulas can identify MetS and might have a better predictive ability compared to other indices. In previous studies, VAI was reported to have good predictive power (identical areas under the ROC curve), which is in agreement with our study findings. However, neither Deuremberg nor CUN-BAE have been assessed. The CUN-BAE equation takes into account age, sex and BMI. These variables are well established as consistent cardiometabolic risk factors, which could support their usefulness in diagnosing MetS. The clinical significance of an adiposity index depends primarily on its ability to predict obesity-related morbidity.

### Strengths and limitations

The main limitation of our study is its cross-sectional design, which does not allow causal relationships to be established, so no conclusions can be drawn about changes in anthropometric measurements over time. Secondly, the population of this study was ethnically homogeneous, as all patients in this study were Spanish population, which could limit the generalizability of our findings. It should be kept in mind that, among representatives of different populations, there are differences in height and body proportions, ethnicity, sex, age, lifestyle, socioeconomic factors, and comorbidities, which may change the relationship between body fat indicators and MetS risk.

One of the strengths of this study is the representativeness of the sample of the adult population of Spain, 418,343 workers, 172,282 women and 246,061 men. Secondly, having evaluated 15 different formulas for the diagnosis of obesity in relation to the 3 main definitions of MetS.

## Conclusions

The anthropometric indices that show the greatest utility in the identification of MetS in our population are the CUN-BAE and the Deuremberg, followed by the VAI. A single definition of MetS needs to be agreed upon in order to determine the best anthropometric index with predictive capacity for its diagnosis.

## Data Availability

Data available on request due to restrictions (privacy or ethical); contact the corresponding author. The head and custodian of the database is Dr. Ángel Arturo López González. Access to information is restricted to the study doctor/collaborators, health authorities, the Research Ethics Committee of the Balearic Islands and authorized personnel, when required to check the data and procedures of the study, but always maintaining the confidentiality of the same in accordance with current legislation. Only the essential data necessary to carry out the study will be transmitted to third parties and other countries, and in no case will they contain information that can identify any patient. In the event that this transfer occurs, it will be for the same purposes of the study described and guaranteeing confidentiality with at least the level of protection of current legislation in Spain.

## References

[1] Ching YK, Chin YS, Appukutty M, Gan WY, Chan YM (2020). Comparisons of conventional and novel anthropometric obesity indices to predict metabolic syndrome among vegetarians in Malaysia. Sci. Rep..

[2] Fernández-Berges D, Cabrera A, Sanz H, Elousa R, Guembe MJ, Alzamora M (2012). Síndrome metabólico en España: Prevalencia y riesgo coronario asociado a la definición armonizada y a la propuesta de la OMS. Estudio DARIOS. Rev. Esp. Cardiol..

[3] Mindikoglu AL, Abdulsada MM, Jain A, Jalal PK, Devaraj S, Wilhelm ZR, Opekun AR, Jung SY (2020). Intermittent fasting from dawn to sunset for four consecutive weeks induces anticancer serum proteome response and improves metabolic syndrome. Sci. Rep..

[4] McCracken E, Monaghan M, Sreenivasan S (2018). Pathophysiology of the metabolic syndrome. Clin. Dermatol..

[5] Wu Y, Li H, Tao X, Fan Y, Gao Q, Yang J (2021). Optimised anthropometric indices as predictive screening tools for metabolic syndrome in adults: A cross-sectional study. BMJ Open.

[6] Riutord Sbert P, Riutord Fe B, Riutord Fe N, Arroyo Bote S, López González AA, Ramírez Manent JI (2022). Influence of physical activity and mediterranean diet on the values of different scales of overweight and obesity. Acad. J. Health Sci. (Medicina Balear).

[7] Matsuzawa Y, Funahashi T, Nakamura T (2011). The concept of metabolic syndrome: Contribution of visceral fat accumulation and its molecular mechanism. J. Atheroscler. Thromb..

[8] Reyes-Barrera J, Sainz-Escárrega VH, Medina-Urritia AX, Jorge-Galarza E, Osorio-Alonso H (2021). Dysfunctional adiposity index as a marker of adipose tissue morpho-functional abnormalities and metabolic disorders in apparently healthy subjects. Adipocyte..

[9] Bello-Chavolla OY, Antonio-Villa NE, Vargas-Vázquez A, Viveros-Ruiz TL, Almeda-Valdes P (2020). Metabolic Score for Visceral Fat (METS-VF), a novel estimator of intra-abdominal fat content and cardio-metabolic health. Clin. Nutr..

[10] Liu PJ, Lou HP, Zhu YN (2020). Screening for metabolic syndrome using an integrated continuous index consisting of waist circumference and triglyceride: A preliminary cross-sectional study. Diabetes Metab. Syndr. Obes..

[11] Bovolini A, Garcia J, Andrade MA, Duarte JA (2021). Metabolic syndrome pathophysiology and predisposing factors. Int. J. Sports Med..

[12] Chiu TH, Huang YC, Chiu H, Wu PY, Clair Chiou HY, Huang JC, Chen SCh (2020). Comparison of various obesity-related indices for identification of metabolic syndrome: A population-based study from Taiwan Biobank. Diagnostics (Basel)..

[13] Bijari M, Jangjoo S, Emami N, Raji S, Mottaghi M, Moallem R (2021). The accuracy of visceral adiposity index for the screening of metabolic syndrome: A systematic review and meta-analysis. Int. J. Endocrinol..

[14] Cho S, Shin A, Choi JY, Park SM, Kang D, Lee JK (2021). Optimal cutoff values for anthropometric indices of obesity as discriminators of metabolic abnormalities in Korea: Results from a Health Examinees study. BMC Public Health.

[15] Kim Y (2016). Han B-G, the KoGES group: Cohort profile: the Korean genome and epidemiology study (KoGES) consortium. Int. J. Epidemiol..

[16] Rabiei N, Heshmat R, Gharibzadeh S, Ostovar A, Maleki V (2021). Comparison of anthro-metabolic indicators for predicting the risk of metabolic syndrome in the elderly population: Bushehr Elderly Health (BEH) program. J. Diabetes Metab. Disord..

[17] Baveicy K, Mostafaei S, Darbandi M, Hamzeh B, Najafi F, Pasdar Y (2020). Predicting metabolic syndrome by visceral adiposity index, body roundness index and a body shape index in adults: A cross-sectional study from the Iranian RaNCD Cohort Data. Diabetes Metab. Syndr. Obes..

[18] Stefanescu A, Revilla L, Lopez T, Sanchez SE, Williams MA, Gelaye B (2020). Using A Body Shape Index (ABSI) and Body Roundness Index (BRI) to predict risk of metabolic syndrome in Peruvian adults. J. Int. Med. Res..

[19] Motamed N, Khonsari M, Rabiee B (2017). Discriminatory ability of visceral adiposity index (VAI) in diagnosis of metabolic syndrome: A population based study. Exp. Clin. Endocrinol. Diabetes Care..

[20] Li R, Li Q, Cui M (2018). Clinical surrogate markers for predicting metabolic syndrome in middle-aged and elderly Chinese. J. Diabetes Investig..

[21] Vinknes KJ, Nurk E, Tell GS, Sulo G, Refsum H, Elshorbagy AK (2017). The relation of CUN-BAE index and BMI with body fat, cardiovascular events and diabetes during a 6-year follow-up: The Hordaland Health Study. Clin. Epidemiol..

[22] Głuszek S, Ciesla E, Głuszek-Osuch M, Kozieł D, Kiebzak W, Wypchło Ł, Suliga E (2020). Anthropometric indices and cut-off points in the diagnosis of metabolic disorders. PLoS ONE.

[23] Suliga E, Ciesla E, Głuszek-Osuch M, Rogula T, Głuszek S, Kozieł D (2019). The usefulness of anthropometric indices to identify the risk of metabolic syndrome. Nutrients.

[24] Davila-Batista V, Molina AJ, Vilorio-Marqués L, Lujan-Barroso L, de Souza-Teixeira F (2019). Net contribution and predictive ability of the CUN-BAE body fatness index in relation to cardiometabolic conditions. Eur. J. Nutr..

[25] Gomez-Marcos MA, Gomez-Sanchez L, Patino-Alonso MC, Recio-Rodriguez JI, Gomez-Sanchez M (2019). Capacity adiposity indices to identify metabolic syndrome in subjects with intermediate cardiovascular risk (MARK study). PLoS ONE.

[26] Baumgartner RN, Heymsfiled SB, Lichtman S, Wang J, Pierson RN (1991). Composición corporal en personas mayores: Efecto de las estimaciones de criterio en las ecuaciones predictivas. Soy. J. Clin. Nutr..

